# Early outcome-prediction with an automated EEG background trend in hypothermia-treated newborns with encephalopathy

**DOI:** 10.1038/s41390-025-04193-9

**Published:** 2025-06-16

**Authors:** Karla Gonzalez-Tamez, Saeed Montazeri, Johan Ågren, Sampsa Vanhatalo, Lena Hellström-Westas

**Affiliations:** 1 Department of Women’s and Children’s Health, Uppsala University, Uppsala, Sweden; 2 Departments of Clinical Neurophysiology and Physiology, University of Helsinki, Helsinki, Finland

## Abstract

**Background:**

Therapeutic hypothermia is an intervention that improves outcomes and alters early outcome-prediction in infants with moderate-severe hypoxic-ischemic encephalopathy (HIE). This study evaluated the early predictive accuracy of a fully automated continuous EEG background trend, Brain State of the Newborn (BSN) in a regional Swedish cohort of infants with presumed HIE.

**Method:**

The BSN trend characterizes 1-min segments of EEG from zero (inactive) to 100 (continuous) and was generated from aEEG/EEG in 85 infants treated with hypothermia. BSN trajectories were compared in relation to clinical grading of encephalopathy and outcome. Receiver operating characteristics were computed for good (no/mild impairment) and poor outcomes (moderate/severe impairment or death).

**Results:**

During the first 48 h, BSN levels differed significantly between moderate and severe HIE (typical median BSN levels >80 and <40, respectively). The predictive accuracy of BSN was high already at 6 h (AUC 0.84) and at 12 h (AUC 0.91), with corresponding positive predictive values (PPV) > 0.92 for good outcome (cutoff BSN > 80) and PPV > 0.95 for poor outcome (cutoff BSN < 40).

**Conclusion:**

BSN gives a continuous and objective measure of EEG background activity, which is highly predictive of good and poor outcomes already from the first 6–12 h in hypothermia-treated infants with moderate-severe HIE.

**Impact:**

- Brain State of the Newborn (BSN) is a deep learning-based EEG trend displaying electrocortical activity as numerical values. Here we establish that BSN trends over the first 48 h differ between infants with moderate versus severe hypoxic-ischemic encephalopathy (HIE) and is highly predictive of long-term outcome.
- This is the first study applying BSN trends in a cohort of exclusively hypothermia-treated infants, demonstrating its value for outcome-prediction already from the first 12 h after birth.
- The BSN provides a continuous bedside evaluation of brain function that complements the visual aEEG/EEG review and can assist bedside assessments in neonatal intensive care units.

## Introduction

Several studies have demonstrated that early electrocortical background activity, as recorded with electroencephalogram (EEG) and amplitude-integrated EEG (aEEG), is predictive of later outcome already during the first 6 h after birth in asphyxiated infants.^1–3^ The main predictive feature is the rate of recovery of the spontaneous electrocortical activity (i.e. aEEG/EEG background) to the infant’s age-typical patterns. Based on this knowledge, early aEEG and EEG are used for evaluation of brain activity after perinatal asphyxia, and for guiding decisions on whether to initiate or abstain therapeutic hypothermia (TH).^4^ Although TH has been shown to improve outcomes,^5,6^ the intervention also alters the predictive information of the early aEEG/EEG. Consequently, several studies have reported that infants receiving TH may have good outcomes even after a delayed recovery of electrocortical activity until 36–48 h of age.^7,8^

The recently developed BSN (Brain State of the Newborn) creates an automated, deep-learning based, quantitative measure of EEG background activity, ranging from zero (inactive) to 100 (fully continuous), which can be displayed as a bedside trend in aEEG/EEG monitors.^9,10^ The BSN has been validated against visual interpretation of the aEEG/EEG background,^9^ and compared to clinical assessment, aEEG, MRI, and outcomes in infants with different grades of HIE, in cohorts including infants receiving TH as well as mixed populations, including both normothermic and cooled infants.^10–13^

Prior BSN-studies have included re-analysis of EEGs from infant cohorts in different clinical trials and with variable outcome measures. However, it is not known how BSN would perform in a population-based, prospectively collected cohort of infants treated with TH for moderate to severe HIE. The present study aimed to assess how BSN relates to the clinical encephalopathy staging, and how an early BSN during TH could predict long-term outcomes in a population-based cohort of exclusively hypothermia-treated infants.

## Methods

### Data retrieval and collection

Data were collected from a regional population-based cohort of infants with moderate-severe HIE (International Classification of Disease, ICD-10 code 91.6), receiving TH during the time-period 2009–2015. Clinical data were prospectively collected in the Swedish National Quality Register (SNQ) and completed with retrospective analysis of aEEG/EEG. The infants were treated at Uppsala University Children’s Hospital, a regional referral center for TH. The health care region covered (population of ~2.1 million), is characterized by relatively large interhospital distances of 80 to 300 km, from six referring hospitals requiring swift postnatal transportation (mostly by helicopter) for timely initiation of TH.^14^ Therapeutic hypothermia is since 2007 offered to newborn infants (gestational ages 36 weeks and above) with indications of acute severe perinatal asphyxia and at least one of the following criteria (according to The American College of Obstetricians and Gynecologists, ACOG):^15^ umbilical cord/early blood sample pH < 7 or base deficit ≥ 16, Apgar score ≤ 5 at 10 min or need for cardiopulmonary resuscitation at 10 min, followed by symptoms of moderate-severe encephalopathy. The Swedish national guidelines were adopted from previous large randomized controlled trials,^4,16^ but do not require aEEG/EEG prior to TH decision. Accordingly, final decision on TH and grading of HIE was performed with information about delivery, need for resuscitation, early blood gases and signs of encephalopathy on arrival to the cooling centre in Uppsala.

### Subjects and aEEG/EEG recordings

**Fig. 1:**
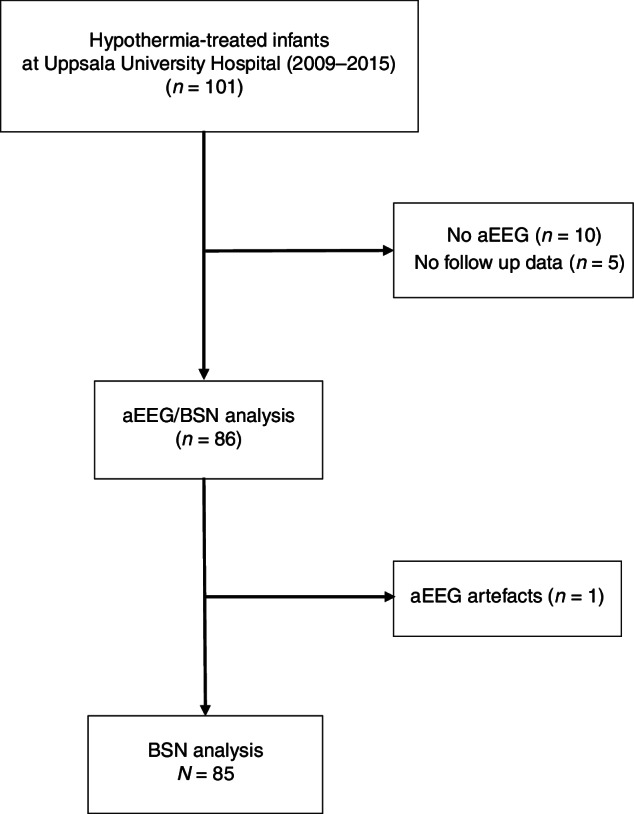
Study population. Presentation of the study population, a cohort of 85 hypothermia-treated newborn infants in a mid-Swedish region.

The study cohort is presented in Fig. 1. The aEEG/EEGs were recorded with NicoletOne™ monitors (Natus Medical Inc., Middleton, WI 53562) mainly with four-disc electrodes, which yielded the following bipolar EEG signals for the BSN analysis: P3-P4, F3-F4, F3-P3 and F4-P4.

The regional research ethics board at Uppsala University approved the study (Dnr 2015/511).

### Clinical outcome categories

Infant outcomes were classified in four categories: 1) survival without any impairment (normal); 2) survival with mild neurodevelopmental impairment (mild NDI), including ambulatory cerebral palsy, Gross Motor Function Classification System (GMFCS) level I-II, or at least one composite (motor, language, cognition) index score 70–84 on the Bayley Scales of Infant Development-III (BSID-III); 3) survival with moderate-severe NDI, including cerebral palsy with GMFCS level III-V or a composite BSID-III < 70; and 4) deceased (Table 1).^17^ Outcomes were also analyzed after further dichotomized grouping as either “good” (normal outcome or mild NDI), or “poor” (survival with moderate-severe NDI, or deceased).

**Table 1:** Definitions^a^ of neurodevelopmental impairment (NDI).

*No NDI*	Developmental milestones met for age (by pediatrician and/or physiotherapist, and/or psychologist)
	BSID III composite^b^: Described as average or above, or Index > 85.
*Mild NDI*	
Motor	Mild delay/difficulties in gross motor skills such as balance, throwing or catching ball.
	Mild difficulties in fine motor skills such as drawing or using a spoon. Cerebral palsy GMFCS level I-II (ambulatory)
Cognitive	Delayed language development or verbal communication, e.g. <25 words.
	Difficulty in putting together two-word sentences.
	Limited non-verbal communication such as body language, eye contact.
	Mildly limited social interaction, or lack of imagination, e.g. in role-play.
	Mild behavioral deficiencies in attention and perseverance.
	May require extra support in daycare compared with peers.
Sensory	Hearing impairment without need for aid, or with adequate correction.
	Visual impairment without need for aid, or with adequate correction.
BSID III^b^	Composite: Described as low average, or Index 70–84.
*Moderate to severe NDI*	
Motor	Moderate or severe difficulties in gross and/or fine motor skills
	Cerebral palsy diagnosis GMFCS level III-V (transitional/non ambulatory)
Cognitive	Moderate or severe cognitive delay e.g., does not understand simple instructions.
	Severely limited verbal, non-verbal communication, attention, or social interaction.
	Autism spectrum disorder diagnosis, severe behavioral issues
Sensory	Uncorrectable hearing loss or deafness
	Visual acuity <0.3, or blindness
Epilepsy	Any diagnosis or treatment
BSID III^b^	Composite: Described as borderline or extremely low, or Index < 70.

^a^Adapted from: Söderström et al. Arch Dis Child Fetal Neonatal Ed, 2021. 106 (4): p. 413–417. (17).

^b^BSID III: Bayley Scales of Infant Development, using the dominant assessment category (Cognitive, language and motor).

### Computation of BSN and analysis of the BSN trends

The BSN trend represents a continuous numerical value of the EEG background activity, derived from a deep-learning based classifier,^9^ unlike prior classifiers that provided discrete results.^18–20^ The range of BSN values from zero to 100, correspond to recovery of an inactive EEG to an increasingly continuous activity, transitioning through burst suppression.^9^ It is important to consider BSN as a continuous value instead of discrete categories. As a rough comparison, however, prior studies^10^ have reported that very low BSN values (from 0 to ~30) correspond to inactive EEG, and relatively low BSN (~30 to ~50) is seen with burst suppression, while modestly lowered BSN (~50 to ~80) relates often to a discontinuous trace, and high BSN (above ~80) reflects a continuous and normalized EEG activity.

The BSN analysis is fully automated and accessible through a cloud service (https://www.babacloud.fi), requiring no specialized equipment or computational expertise. Here, the data was first exported from the Nicolet system and converted to a generic EDF format (European Data Format). The automated system processes EDF-files of raw EEG data (saved in the montage of choice), computing channel wise BSN values for each 1-min of EEG signal,^9^ as well as detecting artifacts and seizures^21,22^ in each channel for post-processing.

From the initial 1-min segments, we computed 1-hourly median BSN values after exclusion of segments with artifacts and seizures lasting longer than 30 s and 6 s, respectively. Individual 1-h epochs were discarded if more than 50% of their segments were excluded.

**Fig. 2:**
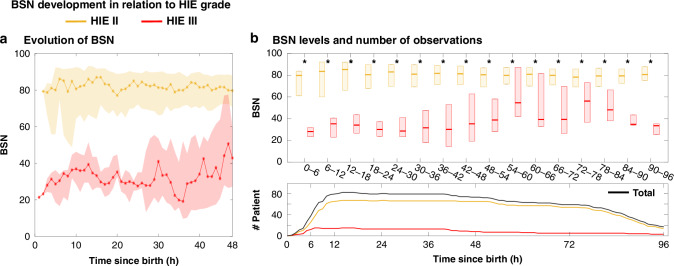
Development of BSN levels over the first 48 and 96 h (medians, Q1 and Q3), for infants with moderate HIE (II) (yellow) and severe HIE (III) (red). **a** BSN values for each 1-h epoch during the first 48 h. **b** Boxplots for the first 96 h, categorized in 6-h epochs and, below, the number of assessed recordings at each time-point: (black = all infants; yellow = HIE II; red = HIE III). *Indicates the rejection of the null hypothesis of equal medians at 5% significance level using Wilcoxon rank sum test.

**Fig. 3:**
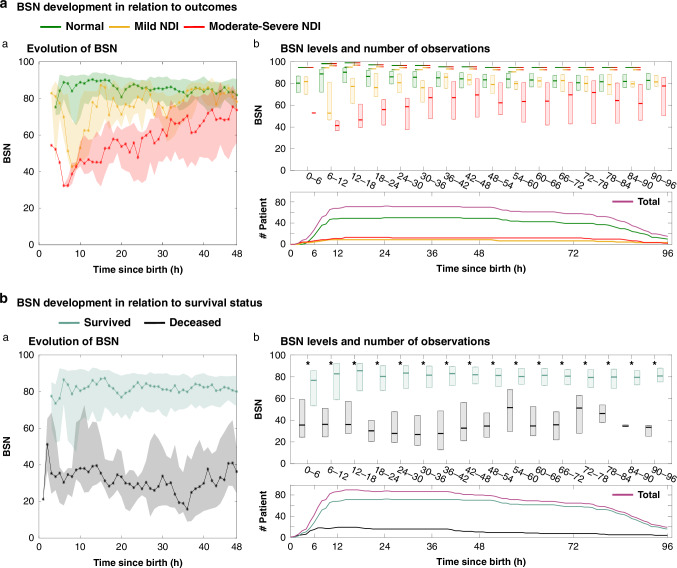
Evolution of BSN over the first 48 and 96 h (medians, Q1 and Q3) in relation to outcome. **a** (**a**) BSN-values (1-h epochs) for surviving infants with normal (green), mild NDI (yellow) and moderate-severe NDI (red) outcomes, and (**b**) 96 h (6-h epochs). The horizontal color-gradient bars over the boxplots indicate group differences with *p* < 0.05 and, below, the number of assessed recordings at each time-point: (fuchsia = all infants; green = normal; yellow = mild NDI; red = moderate-severe NDI outcomes. **b** (**a**) Surviving (green) versus deceased (gray) infants during the first 48 h (1-h epochs) and (**b**) 96 h (6-h epochs). The * over the boxplots indicate group differences with *p* < 0.05 and, below, the number of assessed recordings at each time-point: (fuchsia=all infants; green =surviving; gray=deceased).

All available BSN trends were calculated for each infant for the first 96 postnatal hours, and group comparisons were made between: infants with moderate (grade II) HIE and severe (grade III) HIE (See results Fig. 2a, b), and between the outcome categories in surviving infants (normal, mild NDI and moderate-severe NDI) (See results Fig. 3a), and between surviving and deceased infants (See results Fig. 3b). Recovery for special cases with good and poor outcomes are displayed in the Supplementary Figs. S1 and S2.

### Data treatment and statistical analysis

Clinical data are presented as mean (standard deviations, SD) or median (interquartile range, IQR), with group comparisons analyzed using unpaired two-tailed Student’s t-test, or binary Chi-square analysis with Fisher’s Exact test. The associations between BSN and outcome groups are presented as medians (IQR) over 6- and 12-h periods, respectively, and differences analyzed using Wilcoxon rank sum test. A two-tailed p-value of less than 0.05 was considered statistically significant. The prognostic accuracy for the two outcome categories “good” and “poor” was analyzed by calculations of Receiver operating characteristics (ROC), and the area under curve (AUC) from 1-h segments at 6 h (i.e. median BSN values from 5 to 6 h), 12 h (i.e. at 11–12 h), and in the same fashion at 24, 36 h and 48 h. Similarly, the positive and negative predictive values (PPV and NPV), as well as sensitivity and specificity at chosen BSN cut-offs and time points, were calculated for the outcome categories “good” or “poor” and “normal” and “deceased”, separately.

**Fig. 4:**
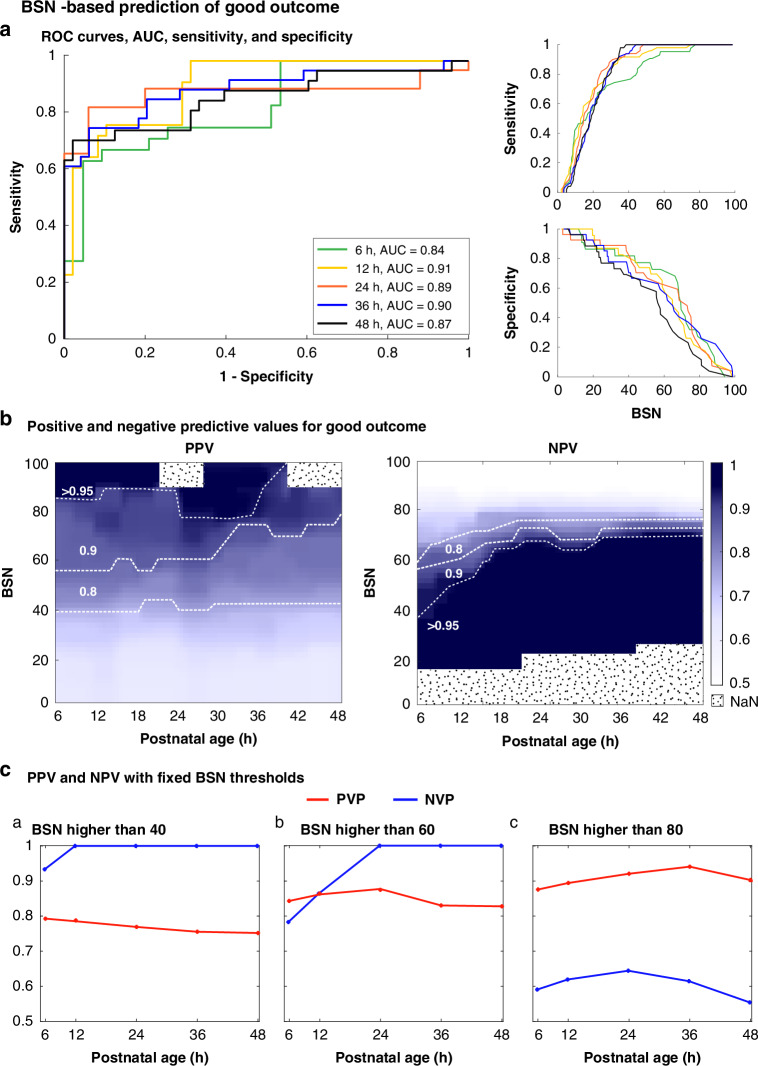
BSN performance for predicting good outcome (normal and mild NDI) (*n* = 85). **a** Left: ROC curves with corresponding AUC values at 6, 12, 24, 36, and 48 h postnatal age. Right: Sensitivity and specificity curves plotted against BSN values. **b** Left: PPV; Right: NPV dynamics, both shown in relation to BSN values and postnatal age (in 6-h intervals). The white dashed lines highlight regions where PPV and NPV fall within the specified value (denoted in white text), corresponding to particular (higher than) BSN values and time intervals. The gradient background color represents the PPV/NPV evolution over time in accordance with the blue-color gradient scale to the right y-axis of NPV (white represents 0 transitioning by a gradient from navy to dark blue, which represents 1). NaN (Not a number) denotes areas where numbers could not be computed. **c** Changes in PPV and NPV across specified time points (6, 12, 24, 36, and 48 h postnatal age) at fixed BSN thresholds (a:40, b:60, and c:80) for good outcome. Notice that a BSN higher than 80 gives a steady PPV of 0.93 from 6 h until 36 h of postnatal age.

The use of the binary outcome categories “good” and “poor” make the results of the outcome predictions complementary, and the ROC and AUC become identical for predicting good and poor outcomes, whereas PPV for good outcomes equals NPV for poor outcomes and vice versa (see Results below, Fig. 4 and Fig. S3). The landscape of the dichotomized results was further supplemented by adding a graded factor, the BSN cutoff level, which shows how prediction of good and poor outcomes is tightly linked to the temporally changing BSN cutoff levels.

## Results

Of 101 infants admitted for TH, 85 had available aEEG/EEG recordings and follow-up data at 2 years. Sixty-seven (79%) infants were outborn. Sixty-eight of the 85 infants had moderate (grade II) and 17 severe (grade III) HIE (Table 2). The aEEG/EEG recordings started at a median (IQR) postnatal age of 6.6 h (5.1–8.8) when TH was established. The total duration of the analyzed EEG recordings was 6085 h (range 4–143 h). Segments with artifacts (9.2%; 361 h from good outcome, 192 h from poor outcome), or seizure detections (1.8%; 52 h from good outcome, 59 h from poor outcome) were excluded, resulting in 5421 h of EEG data reported in the results. Most infants had at least 48 h of aEEG recording (Figs. 2a and 3(a)-b, 3(b)-b, lower right diagram). After artefact rejections, the proportion of infants available for the AUC analyses ranged from 93% (at 6 h) to 98–100% (at 24–48 h) of the total cohort at the given time point.

**Table 2:** Characteristics of the study cohort.

*Study cohort*	*All infants*	*HIE II*	*HIE III*	*p value*
	*N* = 85	*n* = 68	*n* = 17	
Outborn, *n* (%)	67 (79)	55 (81)	12 (71)	ns
Female, *n* (%)	39 (46)	32 (47)	7 (41)	ns
Gestational age, weeks	40.2 (1.4)	40.2 (1.5)	40.2 (1.0)	ns
Birth weight, kg	3.8 (0.6)	3.8 (0.6)	3.6 (0.5)	ns
5-min Apgar score	2.6 (1.8)	3 (1.7)	1 (1.2)	***
10-min Apgar score	3.6 (2.1)	4 (1.9)	2 (1.6)	**
Lowest pH	7.01 (0.03)	7.01 (0.03)	7.00 (0.00)	ns
Highest BD, mmol/L	19.5 (5.6)	17.8 (5.1)	23.7 (4.6)	**
Age at start TH, h	3.8 (1.7)	4.0 (1.7)	2.9 (1.1)	*
Seizures on aEEG/EEG, n (%)	33 (39)	27 (40)	6 (35)	ns
*Outcome*				
Normal, *n* (%)	46 (54)	46 (68)	0 (0)	***
Mild NDI, *n* (%)	8 (9.5)	8 (12)	0 (0)	ns
Moderate/Severe NDI, *n* (%)	12 (14)	11 (16)	1 (6)	ns
Died, *n* (%)	19 (22)	3 (4)	16 (94)	***
Good outcome, *n* (%)	54 (64)	54 (79)	0 (0)	***
Poor outcome, *n* (%)	31 (37)	14 (21)	17 (100)	***

Values are mean (SD), or *n* (%). Good outcome = Normal outcome and Mild NDI, Poor outcome = Moderate/Severe inclusive Deceased.

*HIE* Hypoxic Ischemic Encephalopathy, *TH* therapeutic hypothermia, *BD* base deficit, *HIE* moderate vs severe (unpaired *t*-test, or Fisher's Exact test).

* = *p* value < 0.05, ** = *p* value < 0.001, *** = *p* value < 0.0001 for differences HIE II vs HIE III.

TH was suspended before 72 h in 13 infants, of which 11 died and two required treatment with extra corporeal membrane oxygenation (ECMO) for persistent pulmonary hypertension.

### BSN trends in relation to HIE grade, and outcome

The BSN trajectories in infants with HIE II vs HIE III (*n* = 85) differed statistically significant for most of the 6-h recording periods (see Fig. 2a, b). A comparable and consistent difference was also seen in the BSN trajectories of the three outcome categories in the surviving infants (Fig. 3(a)), as well as between surviving vs deceased infants (Fig. 3(b)). Significant differences persisted throughout the first 36 h between the three outcome-groups of surviving infants with normal, mild NDI and moderate-severe NDI, respectively. After 36 h, the differences in BSN levels became visually more subtle (Fig. 3a, b), although statistically significant differences persisted between the outcome categories, normal vs moderate-severe NDI, mild vs moderate-severe NDI, and survived vs deceased).

### BSN and prediction of long-term outcomes

The predictive accuracy of the BSN trends was assessed using dichotomic outcomes “good” (survival with normal or mild NDI) and “poor” (survival with moderate-severe NDI or death).

The ROC graphs in Fig. 4a and Fig. S3A show the highly predictive accuracy that was obtained already at 5–6 h after birth (AUC = 0.84) (*n* = 33), peaking between 12 h (AUC = 0.91; 95% CI, 0.84–0.97) (*n* = 84) and 24 h after birth (AUC = 0.89; 95% CI, 0.76–0.97) (*n* = 77).

Extracting the sensitivity and specificity curves taken from the ROC analyses suggested clear data-derived cut-off levels for the BSN-based predictions (Fig. 4a and S3A, right side). The sensitivity for predicting a good outcome increased rapidly when BSN increased from 40 to 80 at 6 and 12 h of postnatal age (Fig. 4a). The same curves also indicate specificity for predicting poor outcome (Fig. S3A). Additional ROC curves and AUC estimates for the other outcome categories (normal and death) are presented in Fig. S5.

### Mutual dependence of outcome prediction, postnatal age, and BSN cut-off level

The three-dimensional Fig. 4b and S3B show the detailed, dynamic relationships between PPV and NPV when considering the time from birth and the BSN cutoff levels. The early BSN trend resulted in high predictive values for both good and poor outcomes, however, at different BSN cutoff levels. For instance, a BSN > 80 from start of recording until 36–42 h of age had a PPV above 0.95 for predicting a good outcome (Fig. 4b). In contrast, a BSN < 40 at 12 h, or a BSN < 60–70 after 18 h, had a PPV over 0.95 for a poor outcome (S3B).

### Defining optimal BSN cutoffs for outcome prediction

The 3-dimensional interactions described above and in Fig. 4b and S3B suggest that the optimal BNS cutoffs depend on both the postnatal age and prediction targets. To reduce complexity, we selected three fixed BSN thresholds (a: 40, b: 60, and c: 80) for comparing changes in PPV/NPV over time. For a good outcome, a BSN > 80 gave a steady PPV of 0.91 from 6 to 36 h of postnatal age (Fig. 4c). Correspondingly, the PPV was 1 (NPV 0.8–0.75) for predicting poor outcome at BSN values < 40 from 12 h, or BSN < 60 from 24 h onwards (Fig. S3C).

Supplementary fig. 4 (S4) shows BSN values over time for all deceased infants, including three infants, with initially good brain activity, who died from other causes than HIE (i.e. pulmonary hypertension and multiple organ failure). In Supplementary Figs. 5 and 6 we only show data from infants with normal or death outcomes (mild and moderate outcomes excluded). At 12 h of life, the highest predictive accuracy of a normal outcome was a BSN level above 70 (AUC 0.90) (Fig. S5A). In contrast, a BSN level below 40 (AUC 0.94) at 36 h of age had the highest predictive accuracy for death (Fig. S5B). The BSN cut-off levels for predicting normal outcomes showed a clear variation (range ~80–95) over the first 48 h after birth. (Fig. S6A).

## Discussion

The present study confirms that the BSN trend provides early predictive and clinically useful information in hypothermia-treated infants with moderate to severe HIE. Already from 6–12 h, the BSN trends differed significantly between infants with moderate versus severe HIE and the BSN was also highly predictive of long-term outcomes. The results are in line with other recently published studies reporting differences in BSN levels between infants with different severity of HIE (including mild HIE), and correlations of BSN to later neurodevelopmental outcomes (motor, epilepsy, Bayley scores).^10,11,13^ Our work confirms and extends prior knowledge by showing comparable BSN levels and a high predictive accuracy already during the first 6–12 h after birth, using a population-based cohort of exclusively hypothermia-treated infants with moderate to severe HIE.^23,24^

The correlation between HIE grading by clinical assessment and by EEG trace has been a recognized challenge in previous studies.^2,25–27^ While clinical assessment alone may sufficiently reliable to recognize the most severe cases,^28^ electrophysiological evaluation of cortical function (by EEG or aEEG trend) adds diagnostic accuracy in infants with non-extreme conditions.^29–31^ Our findings are fully in line with prior BSN studies,^11,12^ showing that the BSN trends may effectively distinguish between clinically assessed HIE grades throughout the first 48 h of life.

It is important also to recognize that HIE evolves during the first days after birth, and consequently, it is not reasonable to compare a single perinatal HIE score to a single aEEG/EEG observation at another time point.^2^ In the present study, the postnatal brain recovery from birth asphyxia was very evident in the evolving BSN trends. While many infants with moderate HIE showed a rapid BSN recovery within the first 12–18 h, BSN levels were consistently and demonstrated slower recoveries in the infants with severe HIE. Our findings together with prior studies support the notion that the continuous BSN trend may support a more precise bedside assessment of HIE-recovery during the first two days of life.^10–13^ The practical utility and usefulness of this advancement needs to be assessed in future prospective trials with real-time BSN displayed on bedside monitors.

Several studies have shown that TH apparently delays electrocortical recovery to a continuous background activity, the hallmark of aEEG-based prediction. However, this notion is more likely a misconception since the seemingly delayed recovery also includes infants who were rescued by the TH and therefore recovered later. Nevertheless, the BSN trends can readily and objectively measure differences (“shades of gray”) within each visually identified category, leading to a highly improved discrimination of cortical activity states. Such gradually improving BSN trajectories are readily seen in the present dataset as well, and their significance should be further explored in future studies.

Four recently published clinical studies assessed the utility of BSN in different clinical cohorts.^10–13^ While they all investigated BSN for outcome-prediction, reporting largely comparable results, there were also clear differences between the study cohorts, the inclusion criteria and outcome categories. Some cohorts were retrospective observational collections without specific requirement of hypothermia-treatment^10^ or the cohort could also include infants with mild HIE,^11^ or only perinatal asphyxia,^12^ or the infants were recruited for other purposes in the first place.^13^ The present cohort compares well with the study by Kota et al.^11^ although their population included both normal infants, infants with mild HIE and asphyxiated cooled infants, with start of aEEG monitoring within 6 h. The objective of that study was to assess very early BSN-based prediction and correlation to the HIE grading. Our outcome categories were not fully standardized, but they included clinically meaningful, global and functional outcomes such as NDI, epilepsy and death. The study by Montazeri et al.^10^ showed largely comparable prediction performance with somewhat differing functional outcomes categories. While Lagace et al.^13^ studied mainly hypothermia-treated infants, they also included cases with mild HIE cases. Even though they showed a similar accuracy for BSN predicting “severely impaired” outcomes, the prediction was specifically high for the motor domain at 24 h (AUC 0.97) but a bit lower for cognitive and language outcomes.

In the present study, infants with normal and mild NDI outcomes had rapidly increasing BSN values throughout the first 12 and 36 h, respectively, whereas the BSN in infants with moderate to severe NDI outcomes maintained severely depressed until 48 h, and never really caught up with the normal and mild outcomes groups during the first 96 h. It seems likely that the BSN trend in TH-treated infants may be more sensitive than the aEEG in detecting brain-recovery from a very early age (6–12 h) until 96 h. This could be due to the sensitivity of the BSN trends to depict gradual cortical recovery even within each aEEG/EEG background category. Moreover, the predictive accuracy of BSN at 12 h in the present study compares to the prediction provided by background pattern-based visual assessment of the aEEG at 36 h.^23,24^ The early and precise accuracy of the BSN for outcome-prediction could potentially be of value for future studies, e.g. for decisions on adjuvant therapies to infants treated with TH, or to provide very early outcome surrogates of cerebral recovery, but these need to be established in further prospective studies. It should also be recognized that the BSN-trend does not depict seizures, which calls for a parallel review of the aEEG trend and/or EEG recordings.

Regarding optimally predictive BSN cutoffs, our present study comply with the previous reports on PPV/NPV at 12 h,^10,11^ showing that a BSN > 80 predicted favorable outcomes and <35 predicted CP and death. Kota et al.^11^ also demonstrated that a BSN level of 85 was the most discriminative value between HIE and normal or non-HIE, while a BSN level of 68 was the most discriminative value between mild vs moderate-severe HIE. However, the 2- and 3-dimensional analyses in our work emphasize the rapid dynamics over time between BSN cutoffs vs outcome-prediction (Fig. 4 and S3B, C). Our results may guide the future evidence-based use of BSN in prospective clinical trials, both for patient stratification and for use as a very early surrogate outcome measure.

Our study has three main limitations. Firstly, due to the retrospective nature of data collation, the outcome categories are not fully standardized, however the presently used clinical-functional categorization may support a more holistic and intuitive interpretation of the outcomes. Secondly, BSN trend does not detect seizures per se, though the algorithm has an internal seizure detection to avoid confusions. An additional seizure detection algorithm is needed for guiding anti-seizure treatment. Thirdly, our findings from a single center support the need of further external validation from different centers, and preferably using different EEG systems, before BSN can be implemented in clinical practice; even if the comparable grade of outcome-prediction in previous BSN studies using different aEEG monitors or number of electrodes, could mean that BSN would harmonize aEEG review across different center.^10,11,13^ Nevertheless, a clear strength of our study is the large dataset from very early hours of life in a cohort of exclusively hypothermia-treated infants.

## Conclusions

BSN is a novel sensitive and automated EEG trend to measure brain activity and visualize cortical recovery, to distinguish between infants with moderate and severe HIE, and to very early predict the clinical course of infants treated with therapeutic hypothermia. The BSN outcome prediction varies over time, it depends on the BSN cutoffs as well as prediction targets. Therefore, different clinical situations will need specifically adjusted BSN thresholds and time points for assessment. Our present data shows, however, that the BSN trend provides an accurate very early prediction of long-term outcomes in hypothermia-treated infants throughout the first 48 h of postnatal age, especially for good and poor outcomes already by 6–12 h.

Taken together, BSN trend provides a promising bedside tool for prognostication of outcome, as well as for guiding other decision-making processes of asphyxiated infants in clinical practice. The automated BSN analysis can also aid in supporting further clinical trials for more comparison and harmonizing clinical practices across centers. Further research is planned in this area to expand the knowledge in other BSN applications.

## Supplementary information

Supplementarymaterial

## Data Availability

The datasets generated during the current study are available from the corresponding author on reasonable request.
