# Supplementary material for: Early outcome-prediction with an automated EEG background trend in hypothermia-treated newborns with encephalopathy
Source: Pediatr Res. 2025 Jun 16;99(2):710–7. doi: 10.1038/s41390-025-04193-9 (PMC12956592; doi:10.1038/s41390-025-04193-9)

Figure S1. BSN traces of good brain background activity recovery

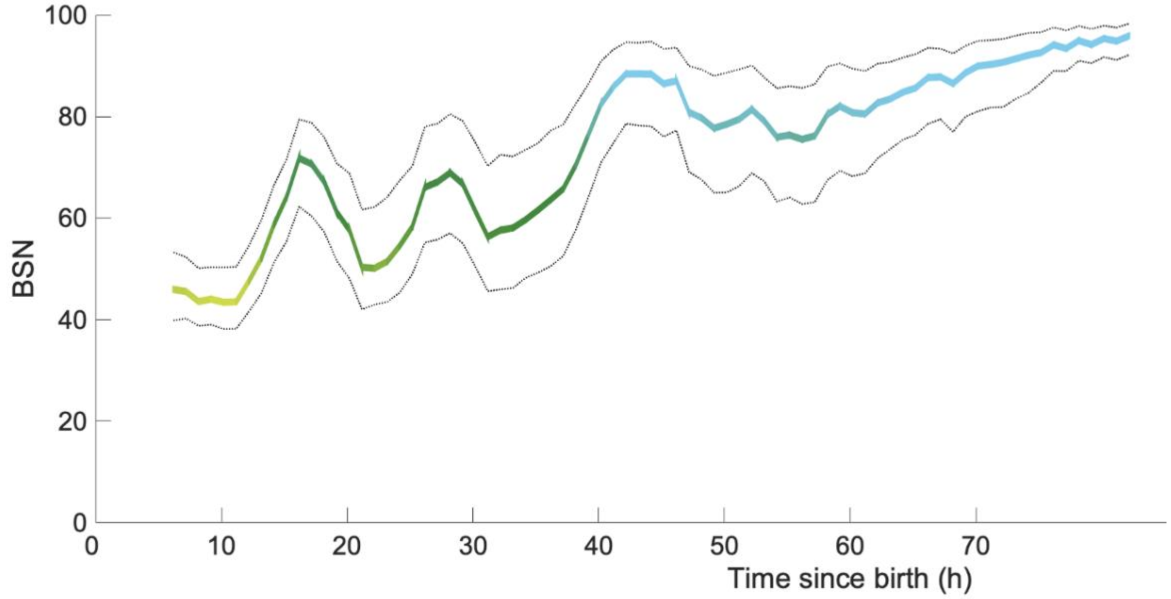

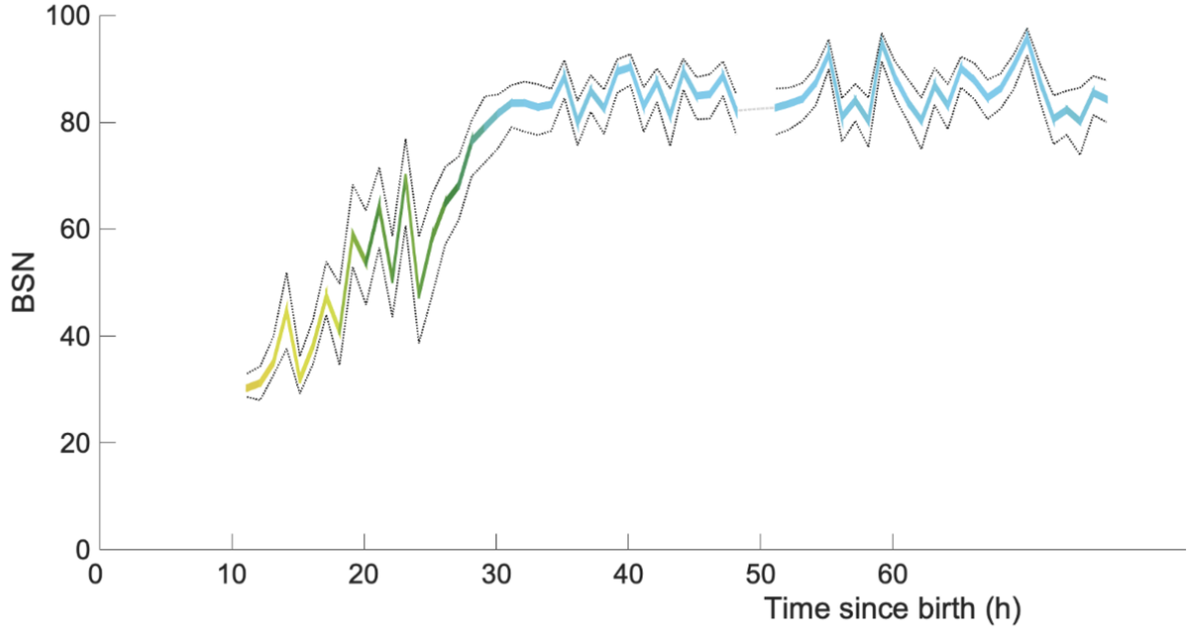

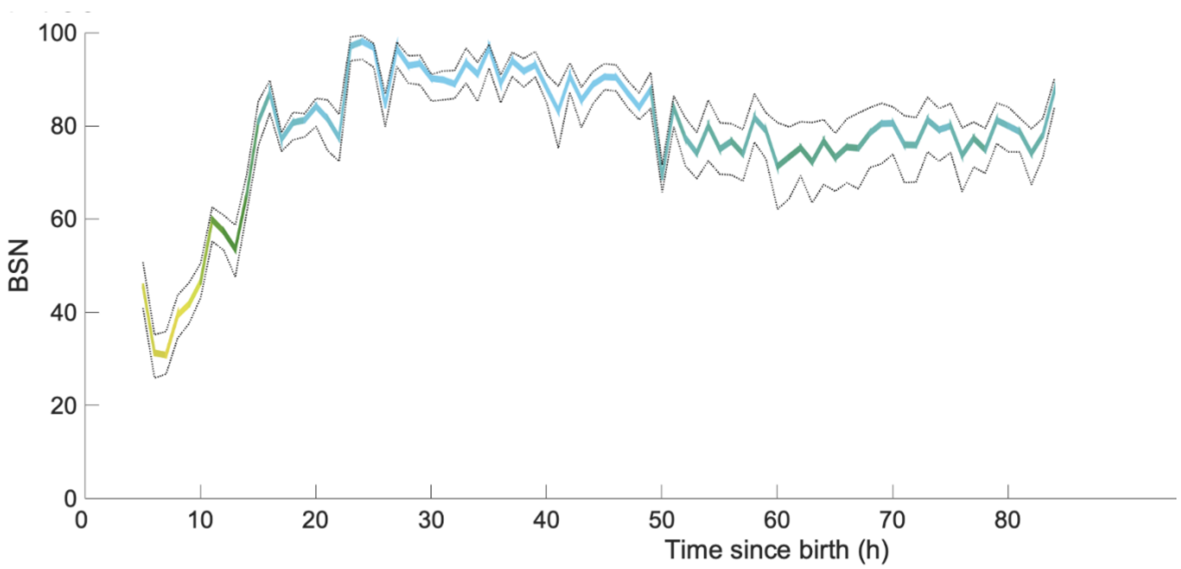

100
80
60
40
20
0
BSN
0
10
20
30
40
50
60
70
80
Time since birth (h)

Figure S2. BSN traces of poor brain background activity recovery

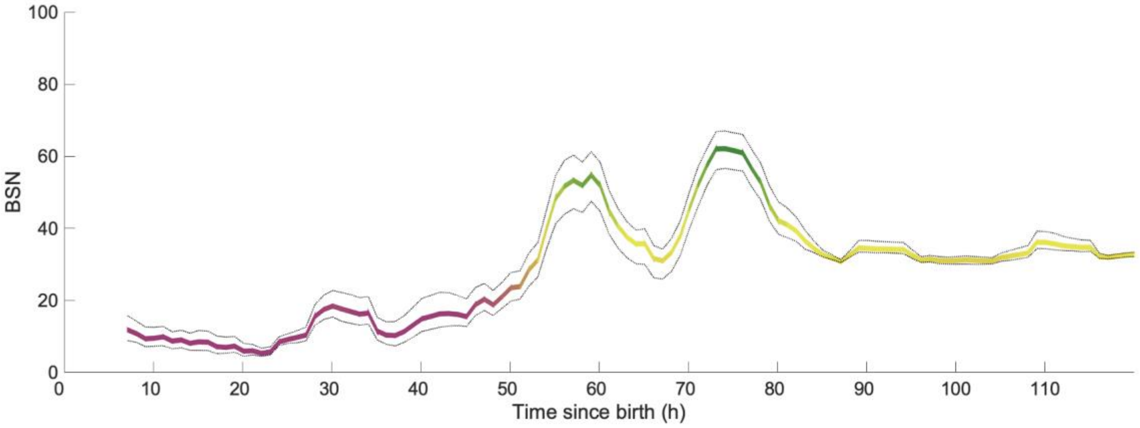

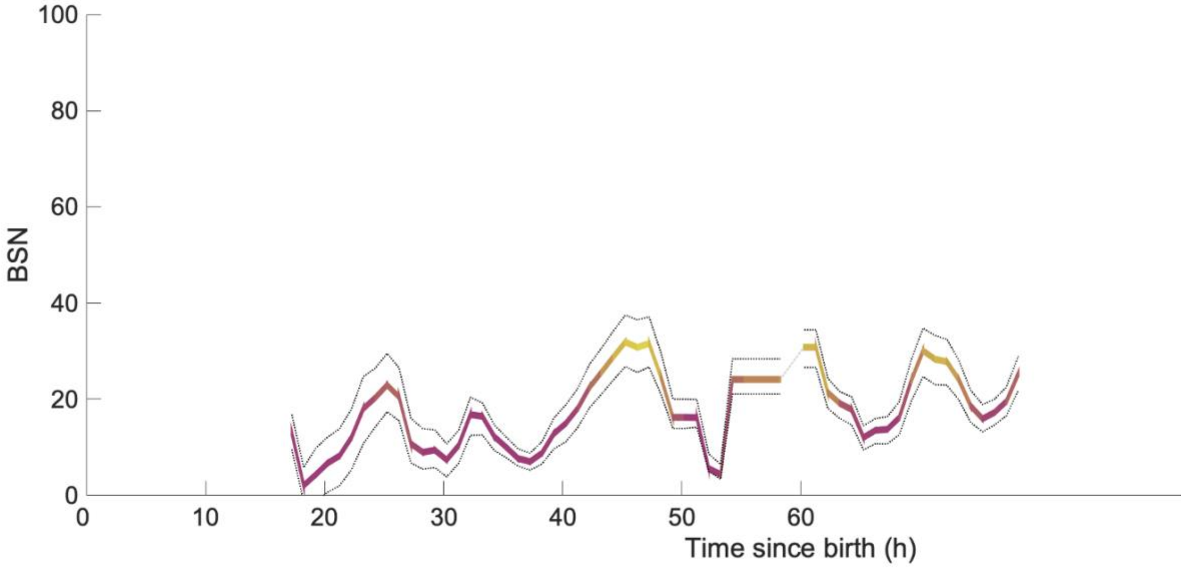

Figure S3. BSN performance for predicting poor outcomes **(moderate/severe NDI and death) (n=85).**

A. Left: ROC curves with corresponding AUC values at 6, 12, 24, 36, and 48 hours postnatal age. Right: Sensitivity and specificity curves plotted against BSN values.

B. Left: PPV; Right: NPV dynamics, both shown in relation to BSN values (y-axis) and postnatal age (x-axis in 6-hours intervals). The white dashed lines highlight regions where PPV and NPV fall within the specified value (denoted in white text), corresponding to particular (lower than) BSN values and time intervals. The gradient background color represents the PPV/NPV evolution over time in accordance with the blue-color gradient scale to the right y-axis of NPV (white represents 0 transitioning by a gradient from navy to dark blue, which represents 1). NaN (Non a number) denotes areas where numbers could not be computed. The NPV dynamics for poor outcome are the same as the PPV dynamics for good outcome and, respectively, the PPV dynamics for poor outcome are the same as the NPV dynamics for good outcome (***see also Figure 4).***

C. Changes in PPV and NPV across specified time points (6, 12, 24, 36, and 48 hours postnatal age) at fixed BSN thresholds (a:40, b:60, and c:80) for poor outcome. Notice that a BSN lower than 40 gives a steady PPV of 1 from 12 hours of postnatal age.

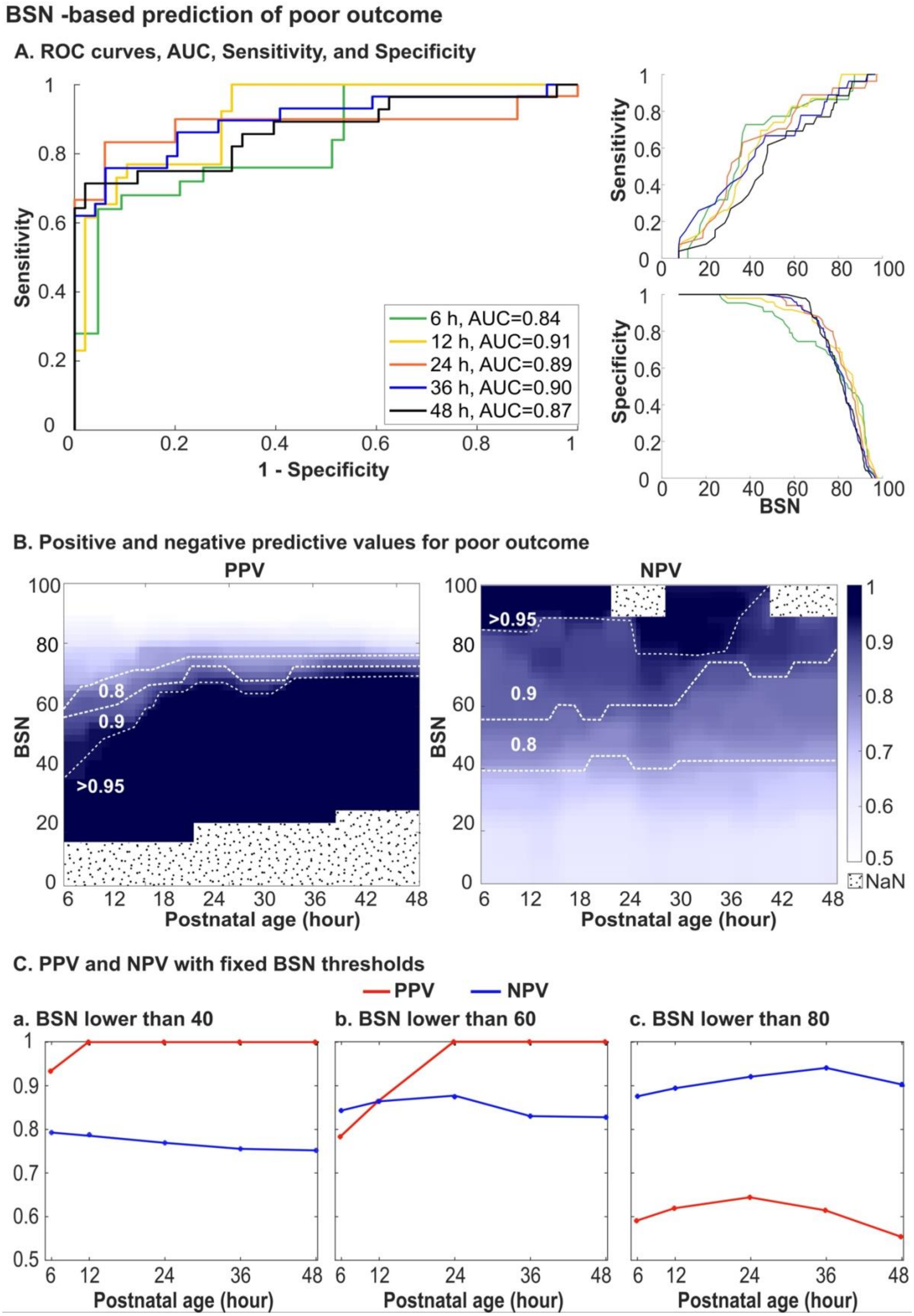
BSN -based prediction of poor outcome
A. ROC curves, AUC, Sensitivity, and Specificity
Sensitivity
1 - Specificity
6 h, AUC=0.84
12 h, AUC=0.91
24 h, AUC=0.89
36 h, AUC=0.90
48 h, AUC=0.87
Sensitivity
Specificity
BSN
B. Positive and negative predictive values for poor outcome
PPV
NPV
BSN
0.8
0.9
>0.95
Postnatal age (hour)
NaN
C. PPV and NPV with fixed BSN thresholds
PPV
NPV
a. BSN lower than 40
b. BSN lower than 60
c. BSN lower than 80
Postnatal age (hour)

Figure S4. The individual BSN development in all deceased infants.
The red rings mark three outliers, one infant died of multiple organ failure, and the other two of persistent pulmonary hypertension. The clinical interpretation of the initial aEEG recordings in these cases were CNV/DC, supporting the notion that these infants had a favourable brain activity before their conditions deteriorated.

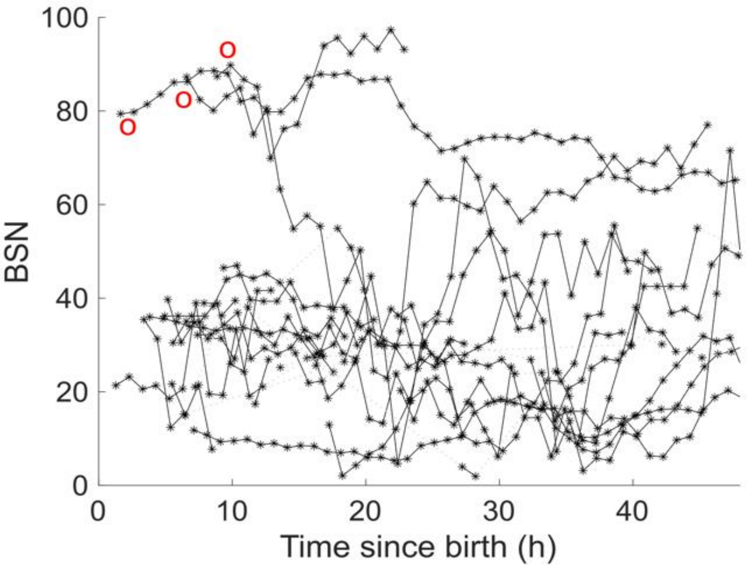

Figure S5. Performance of BSN, additional predicting outcomes.
A. Left: ROC curves for **Normal** outcome, with corresponding AUC values at 6, 12, 24, 36, and 48 hours postnatal age. Right: Sensitivity and specificity curves plotted against BSN values. **B**. Left: ROC curves for **Death** outcome, with corresponding AUC values at 6, 12, 24, 36, and 48 hours postnatal age. Right: Sensitivity and specificity curves plotted against BSN values.

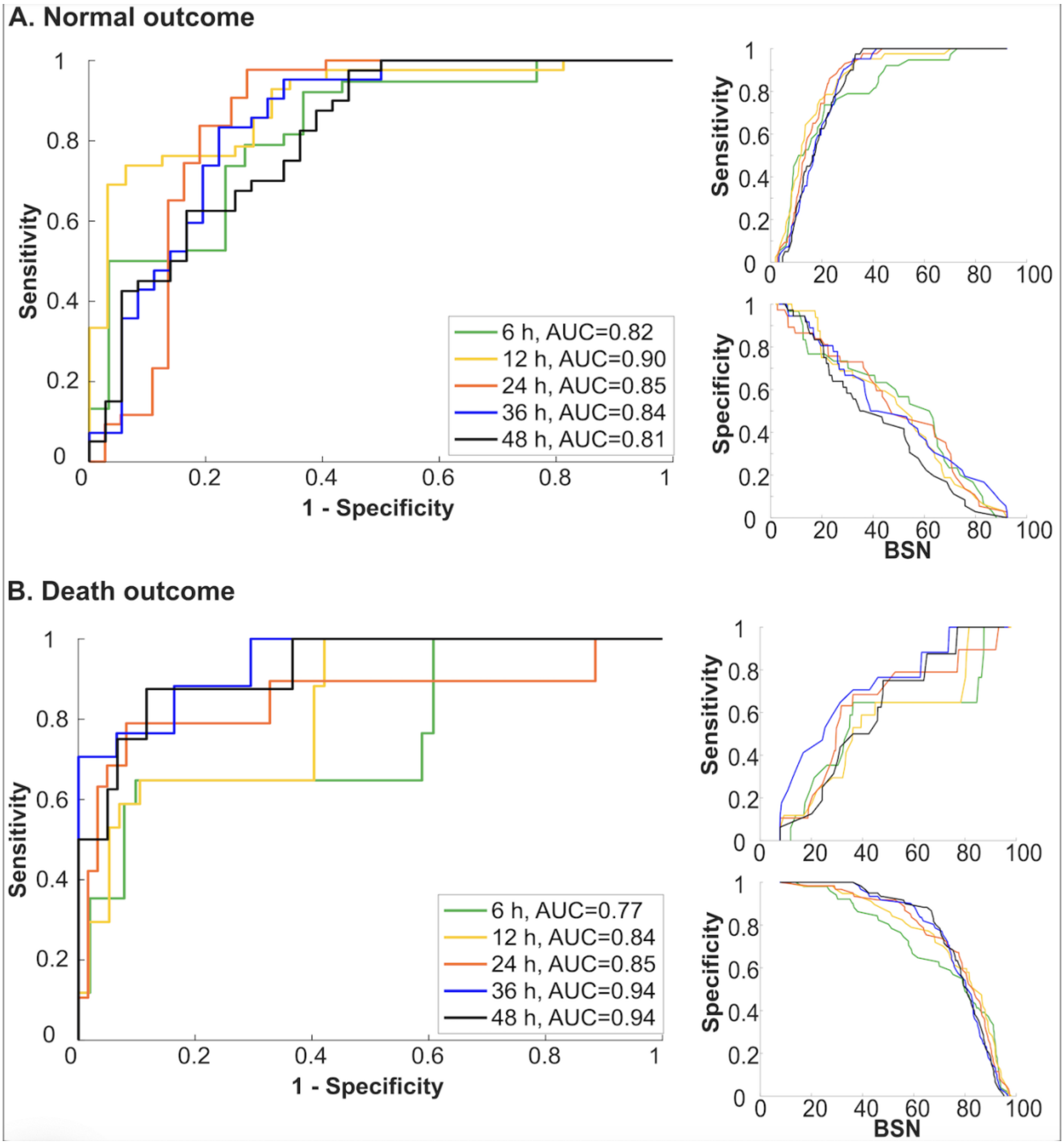

Figure S6. Prediction outcomes by PPV and NPV for **(A.)** Normal outcome and **(B.)** Death outcome.Top: PPV; Bottom: NPV dynamics, both shown in relation to BSN values (y-axis) and postnatal age (x-axis in 6-hours intervals). The gradient background color represents PPV/NPV for the given prediction. White represents 0 and black represents 1. The BSN threshold is marked as a purple line. Optimal BSN value is marked as purple stars in a 6 hours-epoch. NaN (Non a number) denotes areas where numbers could not be computed.

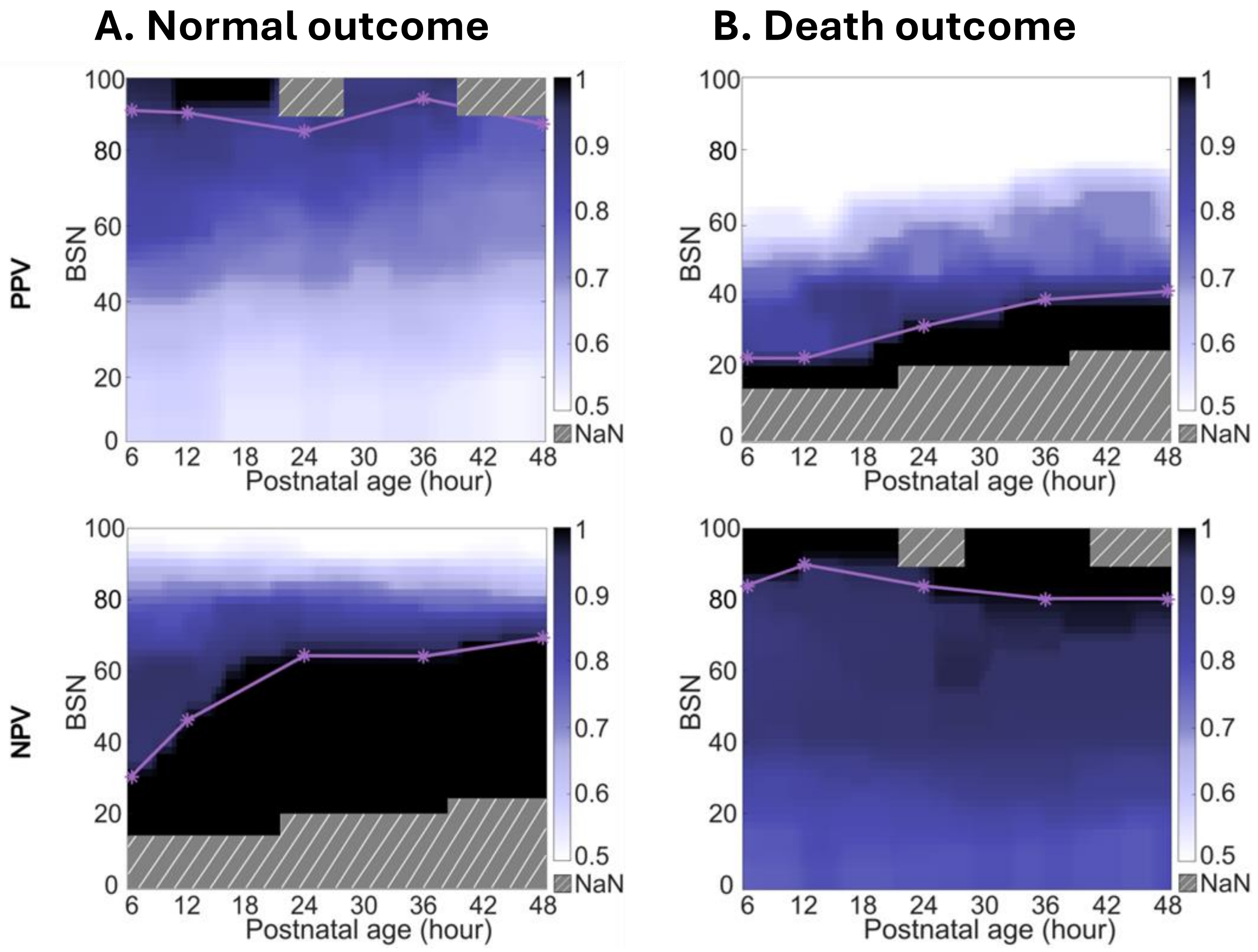

Supplement: Supplementary file 1 — Supplementarymaterial [file 41390_2025_4193_MOESM1_ESM.pdf]
